# Editorial: How Best to “Go On”? Prospects for a “Modern Synthesis” in the Sciences of Mind

**DOI:** 10.3389/fpsyg.2016.00766

**Published:** 2016-05-30

**Authors:** Kevin Moore, John Cromby

**Affiliations:** ^1^Faculty of Environment, Society and Design, Lincoln UniversityChristchurch, New Zealand; ^2^School of Management, University of LeicesterLeicester, UK

**Keywords:** theoretical synthesis, multi-level systems, neurophenomenology, levels of explanation, enactivism, developmental systems theory, reciprocity, argumentation

For some time, conceptual unity in psychology has been seen as both a scientific “holy grail” and a feared hegemonic project—see, for example Observer (1982), Kantor (1984), and Dixon (1983). This may be because a focus on integration, perhaps paradoxically, may intensify various tensions within a psychology whose sub-disciplinary constitution actually reflects fault lines and dualisms in the organization of knowledge more generally.

In recent years, we have seen new areas of theory and methods, including enactivism, embodied cognition, discursive psychology, second-person neuroscience, developmental systems theories, and a stunning growth in the neurosciences, genetics, and epigenetics. Our contributors explore whether such advances have helped synthesize the diverse understandings of mind within psychology. In so doing they frequently emphasize the unifying prospects of dynamic, adaptive, action-orientated, “socialized,” systems-based, and embodied approaches, and are correspondingly critical of reductionist, mechanistic approaches.

The articles are of two kinds. The first deals directly with the integration of the sciences of mind and cognition as a broad project (Marshall; Stam; Andringa et al.). The second focuses on prospects for synthesis within specific contexts of theory, method, and practice, including reciprocity (Berra) psychiatric theory, and diagnosis (Castiglioni and Laudisa; Di Francesco and Marraffa) methods for investigating consciousness (Olivares et al.); theories of vision (Laurent) and argumentation (Lillo-Unglaube et al.).

Marshall's proposal concerns relationships between “levels” in psychological understanding, arguing that combining an embodied approach with a developmental systems account, within a relational worldview, overcomes the conceptual “splitting off” of mind from brain and body. He highlights the vital role that “pattern explanation” (akin to Aristotle's formal cause) has in a relational developmental systems approach, because it allows increased conceptual clarity over the relations between a system's organization and its activity and thus avoids reductionism.

With a similar focus on the integration of levels of explanation, Di Francesco and Marraffa consider the relationship between consciousness (personal level) and the unconscious (sub-personal level). They argue that, contra an eliminativist perspective, some personal level concepts such as “motivation” and “attachment” can, in dialectical relationship with neuroscientific findings, provide a useful indication of how personal and sub-personal levels of explanation can operate together.

Relatedly, Castiglioni and Laudisa reject what they see as the reductionist, biological underpinnings of the DSM-5 approach to psychiatric diagnosis. Their article speaks to a context where consistent evidence for the biological deficits purportedly associated with the functional psychiatric diagnoses remains elusive, and where major funders such as NIMH no longer rely upon these diagnoses. Focusing on the DSM categorization of depressive disorders and the removal of the “bereavement exclusion clause”—and thus the continuing conflation of “endogenous” and “reactive” categories of depression—they show that reducing experiences of distress to quasi-medical symptoms actually undermines theoretical and clinical accuracy.

Digging further into neurological findings, Laurent proposes a new “Multiscale Enaction Model” of visual perception that challenges a hard-wired, modular account. He suggests that converging data indicate a need to acknowledge the multiple systems at various scales that interconnect to create visual experience.

With a similar focus on the relations between neurological findings and phenomenological experience, Olivares et al. examine the potential for “second-person methods” (through interviews) to fulfill the promise of a unified neurophenomenology. They argue that such methods provide more systematic data than do direct first-person methods (e.g., introspection in both its strong and weak forms) and can help bridge experiential and neurological descriptions of conscious experience.

Berra addresses the important phenomena of altruism and reciprocity in primate social groups. The aggregation of individuals into socially-bonded and cooperative groups, she argues, is best explained by emotional tracking of interactions—rather than the more cognitively “expensive” bookkeeping of expectations of rewards suggested in other theories. Her parsimonious approach facilitates consistent explanations of social reciprocity throughout primate groups that exhibit various levels of cognitive capacity. It also suggests a theoretical synthesis of emotional processes with the requirements of complex and dynamic adaptive social behavior.

For humans, cooperation also often involves debates and the making of arguments. As discursive psychology demonstrates, these psychological processes are simultaneously fundamental to political, legal, scientific, and educational discourse. The potential synthesis of normative (“classical”) and cognitive approaches to understanding human argumentation is investigated by Lillo-Unglaube et al. They examine two argumentative fallacies (the “slippery slope” argument, and the *ad hominem* argument), and conclude that descriptive and experimental studies could potentially integrate normative and cognitive research traditions to produce an integrated body of theory on the psychology of argumentation.

In their ambitious contribution, Andringa et al. provocatively suggest that all cognition derives from two general modes that are based on common tendencies in all life forms: the coping mode, and the co-creation mode. The first is structured around the goal of meeting immediate needs while the second operates to construct environments within which pressing needs are less likely to arise.

The selection concludes with Stam's insightful argument over the very idea of conceptual synthesis in the sciences of mind. He proposes that psychology is in fact already relatively unified *methodologically*, through the adoption of an “indeterminate functionalism.” He then argues that the neurosciences, while not acting to synthesize psychology, will nevertheless, influence our understanding of being human—perhaps by coming to see the brain as a technology that we use, but do not fully understand.

What, then, are the prospects for this “Modern Synthesis,” and how should we best “go on?” Unsurprisingly, these papers provide no unequivocal answer to these questions. What does emerge, however, is that posing them raises many challenges—across theory, method, and practice, and at a range of scales. It is also clear that raising the issue of conceptual synthesis reveals significant bumps and hollows in our understanding of mind, and inspires innovative responses to those challenges.

## Author contributions

KM wrote the first draft of the Editorial. JC revised that draft and the revisions were incorporated into the final manuscript. KM has now revised the manuscript in the light of the Editor's review comments.

### Conflict of interest statement

The authors declare that the research was conducted in the absence of any commercial or financial relationships that could be construed as a potential conflict of interest.
